# Collective empowerment of an online patient community: conceptualizing process dynamics using a multi-method qualitative approach

**DOI:** 10.1186/s12913-021-06988-y

**Published:** 2021-09-13

**Authors:** Marie-Georges Fayn, Véronique des Garets, Arnaud Rivière

**Affiliations:** VALLOREM Lab, University of Tours, 50 Avenue Jean Portalis, 37200 Tours, France

**Keywords:** Collective empowerment, Online community, Patient, Health services

## Abstract

**Background:**

Online communities for patients with chronic conditions are becoming healthcare providers. They gather to offer support and services, and to become a collective oppositional force. We found, however, that these communities and their collective power are rarely studied in the health services management literature, which focuses more on the empowering practices of healthcare professionals or patient participation. The aim of this study is thus to build a better understanding of the nature of patients’ collective empowerment and the processes underlying it. We carry out two exploratory qualitative studies to examine the motivations that drive chronically ill patients to engage in an individual and then collective empowerment process.

**Methods:**

The first qualitative study involves four semi-structured interviews with experts. The second is a netnographic study carried out over a year on an online forum for people with thyroid disease. The latter has two phases: an immersion phase followed by one that traces the path of 21 forum members from their first message to their recognition as active members or even forum moderators. The data are analyzed through thematic and lexical content analyses.

**Results:**

We were able to identify the different stages of the collective patient empowerment process and the criteria for progression though this process. Specifically, the first study sheds light on the unmet individual and collective needs of the patients. The second emphasizes the essential role of active contributors and their impact on the growth and power of the community.

**Conclusions:**

This study looks at patient communities as a self-contained system and identifies the stages of collective empowerment that match the organization’s declared priorities: community, collaborative, productive, and societal. These results should help health professionals better take these online communities into account in patient care, improving their practices, and carrying out their policies. We call for future research into collective empowerment and its influence on patient behavior, the transformation of healthcare institutions, and the health services market.

**Supplementary Information:**

The online version contains supplementary material available at 10.1186/s12913-021-06988-y.

## Background

“*Hi, my TSH* (Thyroid Stimulating Hormone) *is ‘good’, but I don’t feel good! How can I convince my doctor to increase my dosage? He only looks at my TSH rate, but I’ve seen on the forum that you also recommend looking at my low T3 and T4 and my personal set point.”.*^1^ Patients today are no longer isolated and fragile individuals requiring health education programs [1], but are engaged actors, continually interacting with organized, influential, and knowledgeable online communities [2]. Thousands of patient organizations are a source of solidarity and innovation and improve everyday life for the sick by helping them overcome obstacles and fight against inequality and stigma [3, 4]). Thanks to better and more accessible digital platforms, these online communities play an essential and growing role in patients’ individual and collective empowerment, thus becoming a potential player in healthcare system performance [5–7]. Empowerment has in fact been mentioned by health institutions [8, 9] as a key concept that could drive progress in public health and cost savings through more informed consumption of health services [10, 11].

In the health services management literature, empowerment has mainly been addressed in the workplace context from the point of view of organizations and health care teams. Various concepts have been studied from this perspective, including structural empowerment (empowering conditions in the workplace like social workplace conditions and structures and workplace policies), empowering leadership (a set of leader behaviors involving sharing power or allocating more responsibilities and autonomy to followers) and psychological empowerment in the workplace (focusing on the individual and the employees’ motivational state) [12–15]. In contrast, our research considers an alternative approach that focuses on the patient’s point of view and defines empowerment as “a social action process by which individuals, communities and organizations gain mastery over their lives in the context of changing their social and political environment to improve equity and quality of life” ([5], p. 198). This increased power comes through the transfer of information and skills [16]. From this perspective, empowerment can be seen as both a process and a result of the communication between healthcare professionals and patients [17, 18]. Since most of the prior research has been conducted within a patriarchal Western culture promoting individualism, competition, and submission to medical paternalism [19–21], it has presented a limited view of empowerment. First, these studies have taken a top-down approach to this concept, in which healthcare professionals convince patients to be active participants in their treatment [1] and convince the healthy to remain so through a healthy lifestyle. By focusing on the top-down approach of professionals and ignoring any bottom-up dynamics, these studies suffer from their conception of the patient’s role as passive [22], thus underestimating patients’ psychological resources and interpersonal and collaborative skills [23–25]. Moreover, academic research has given pride of place to the study of psychological empowerment by defining the components of this mental state [26], especially patients’ ability to take charge of their fate, mobilize their resources, and act to transform their environment [27, 28]. Still, a process approach to empowerment has seen relatively little interest from researchers. Finally, most of the studies have chosen an individual, patient-centered approach [29] at the expense of a deeper understanding of their collective being [30, 31], thus setting aside the voluntary process of affiliating with a peer group and tapping into collective intelligence. Given these analyses and considering the new status of citizen-patient as well as the power of connecting with a peer community, it is thus necessary to add depth to the study of empowerment, taking a bottom-up approach that is collective and process-driven.

This research thus looks at the role of online patient communities to build a deeper and better understanding of the complex process of collective empowerment, especially its different phases and criteria for progression through the process. We begin with a literature review based on two approaches: first, how health and social actors transmit skills and knowledge; second, how patient communities have acquired power [3, 4]. Beyond the analysis of various dimensional structures and processes, we also look at the contribution of social media, which has catalyzed collective empowerment [32]. The approaches and results in this review bear witness to the malleability of a continually evolving concept and the limited view of collective empowerment [33, 34]. As this concept has not been thoroughly investigated from the patients’ point of view in the past, and considering the need to gain familiarity, acquire new insights, and obtain a more in-depth and holistic view about the components of collective empowerment within online patient communities [35], an exploratory approach is used. In this light, we carry out two qualitative studies, one of which is netnographic. These studies look at patient communities as collective subjects in their own right. Starting with needs and moving to internal and external interactions, our studies contribute an original understanding of the collective empowerment process. Our results provide a number of theoretical and managerial contributions and give rise to fruitful avenues of research, some of which expand on the limitations of this empirical study.

### Conceptual framework

We have divided the small number of health services management articles devoted to collective patient empowerment into two categories. The first includes top-down empowering practices used by health and social actors as part of regionally funded programs (Table 1). The second category involves research on bottom-up empowerment through online communities of patients with the same ailments and/or social vulnerabilities [43] (Table 2).

**Table 1:** Studies of collective empowerment of communities through empowering practices by health and social actors

	Goals	Approach and context	Contributions to the collective empowerment of the community
Labonte [36]	Help participants engage in empowering actions in communities	Conceptual, personal analysis based on 6 years of training workshops involving 2500 professional communities	Categorizes interactions between professionals and patients that contribute to collective empowerment: • Interpersonal: personal care • Intragroup: support of the organization of peer communities • Intergroup: help building alliances among groups • Interorganizational: support for the movement’s political stances Identification of a dialectic in which power is simultaneously given and taken away
Speer and Peterson [37]	Provide social actors with measures to understand community empowerment and the correlation between psychological empowerment and participation in community activities	Empirical approach through a survey given to 974 members of a community acting to fight drug abuse	Development of an interactional empowerment scale with three dimensions: • Cognitive: understanding the relationship between power and the dominant ideology • Emotional: leadership ability and political influence • Organizational: shared emotional connection and feeling of belonging to a community
Laverack [38]	Better identify and conceptualize the organizational aspects of community empowerment in the context of managing healthcare programs. The goal is to increase the engagement of participants in the development of local communities	Conceptual approach through a literature review	Make the community empowerment process operational in a program framework: 1. Improve participation 2. Increase local leadership 3. Enable cohesion and socialization in organizations 4. Build the ability to evaluate problems 5. Improve the use of resources 6. Improve critical thinking skills 7. Strengthen relationships with other individuals and organizations 8. Create an equitable relationship with external agents 9. Share program management with communities These areas are interdependent.
Wiggins [39]	Build awareness of the effectiveness of popular education to increase empowerment and improve health	Conceptual approach through a literature review	Conception of a three-dimensional model of empowering education: individual, organizational, and community, with an internal or external locus. This nonlinear process varies according to the people, organizations, and communities involved. Different components of psychological empowerment are included in this model: self-esteem and -confidence, critical consciousness, sense of community, increased participation, personal and collective control, acts of solidarity and mutual aid to achieve community goals, and increased motivation to spark change through advocacy
Melo and Alves [40]	Propose a theoretical nursing model (MAIEC) based on community empowerment. This nursing model offers an integrative vision of stakeholders and local structures and provides a clinical decision matrix to guide nurses’ decision-making.	Conceptual approach [40] and empirical approaches through a cross-sectional quantitative study (developed in a community of schools in Africa to address children’s nutritional status and eating behaviors - [41]) and focus groups (with four Portuguese primary healthcare structures to improve the epidemiological monitoring of nursing diagnoses – [42]).	Community empowerment is approached as a dynamic process articulated around three components: - Community leadership related to the community’s knowledge, beliefs, behaviors, and wishes in the context of the problem addressed; - Community participation related to communication, partnerships, and the existence of organizational structures; - Community process related to community coping or experiences with the problem addressed.

**Table 2:** Studies of collective empowerment of online communities through patient participation

	Goals	Approach and context	Contributions to the collective empowerment of the online patient community
Petrič and Petrovčič [44]	Determine to what extent the determinants of individual empowerment are related to collective power	Empirical approach. Pilot study with 270 users of the same forum and then with members of 81 online forums	Identification of congruence between individual and collective empowerment: • Sense of virtual community • Engagement with the organization and the community’s vision • Participation in the daily life, activities, and demonstrations organized by the community through positive interactions The emphasis is on the quality of social interactions, mutual respect, tolerance, and critical understanding.
Ammari and Schoenebeck [45]	Contribute new knowledge on the role of social media in the empowerment of parents of sick children	Empirical approach 43 interviews with parents of children with specific health problems	Introduction of a new network empowerment model Identification of three stages: • Join an online forum after diagnosis • Ask other parents about services • Become advocates for their children’s needs Highlights the role of “veteran” parents who • Advocate at a broader level than just for the needs of their own children • Teach other parents how to mobilize resources • Sometimes educate legislators
Demjén [46]	Study how patients use humor in online discussions of their experiences with cancer.	Empirical approach Analysis of 530,055 words on a thread about humor in an online forum for English cancer patients	Highlights the empowering potential for humor: • Makes it possible to discuss taboo topics • Builds rapport among members • Bonds the community • Affirms the collective power of resistance to a sometimes uncontrollable disease • Gives the feeling of taking control and reacting freely Humor is enabled by the relative anonymity of virtual communities and their constant accessibility.
Atanasova and Petric [47]	Develop a measurement instrument to evaluate the collective empowerment of online patient communities and then test its validity	Empirical approach Pilot study with 280 members of an online patient community in Slovenia and then with about 30,000 members	Creation of a measurement scale for collective empowerment along two dimensions: • Knowledge of resources and methods used to impact change • The mobilization of these resources for collective action Also related: • The sense of virtual community • Engagement in community organization • Intensity of participation • Civic participation • Offline emotional support

#### Top-down approach: a reductive approach to collective empowerment

The first category of research (Table 1) is aimed at health and social practitioners; its goal is to make the notion of community or collective empowerment more operational by simplifying it. These studies all refer to the idea of empowerment moving from the individual to more collective stages. The individual level involves each person’s knowledge, abilities, and competencies. The collective levels are divided into two categories, organizational and community [3]. To explain how communities are empowered, Labonte [36] proposes a “holosphere” composed of five interlocking circles. This overlap illustrates the interdependence of professionals and patients at various levels: interpersonal, in the care provided; intragroup, supporting individuals in forming a group; intergroup, supporting groups in their alliances with other groups; and interorganizational, supporting movements to defend patients at the political level. Speer and Peterson [37] develop an interactional empowerment scale for social workers. Their scale has three components: a cognitive dimension from a critical analysis of the forces that shape the environment; an emotional dimension referring to leadership and political influence; and an organizational dimension involving the sense of community, connection, and belonging. Laverack [38] analyzes the relationships between empowerment and health gains in publicly funded programs. He identifies three stages, from individual empowerment to social and political action and finds a correlation between entry into a collective empowerment process and an increase in psychological empowerment. To extend these results, researchers have looked at the extent to which psychological and collective empowerment reinforce each other [44, 48–50]. Wiggins [39] bases her work on a Freirian view of liberating empowerment [51] and the systems defined by Wallerstein [6]. She illustrates empowering health education as a spiral moving from an individual and internal level to the most collective, external level. The primary components are the sense of community, personal and collective control, mutual aid, the pursuit of community goals, and advocacy for social change. More recently, a theoretical nursing model has been developed (the Community Assessment, Intervention, and Empowerment Model – MAIEC), based upon clinical decision-making for community health nurses using communities as a unit of care. This approach considers community empowerment as a dynamic process combining community participation, partnership, and leadership to identify and solve problems and call upon community resources [40].

**Fig. 1:**
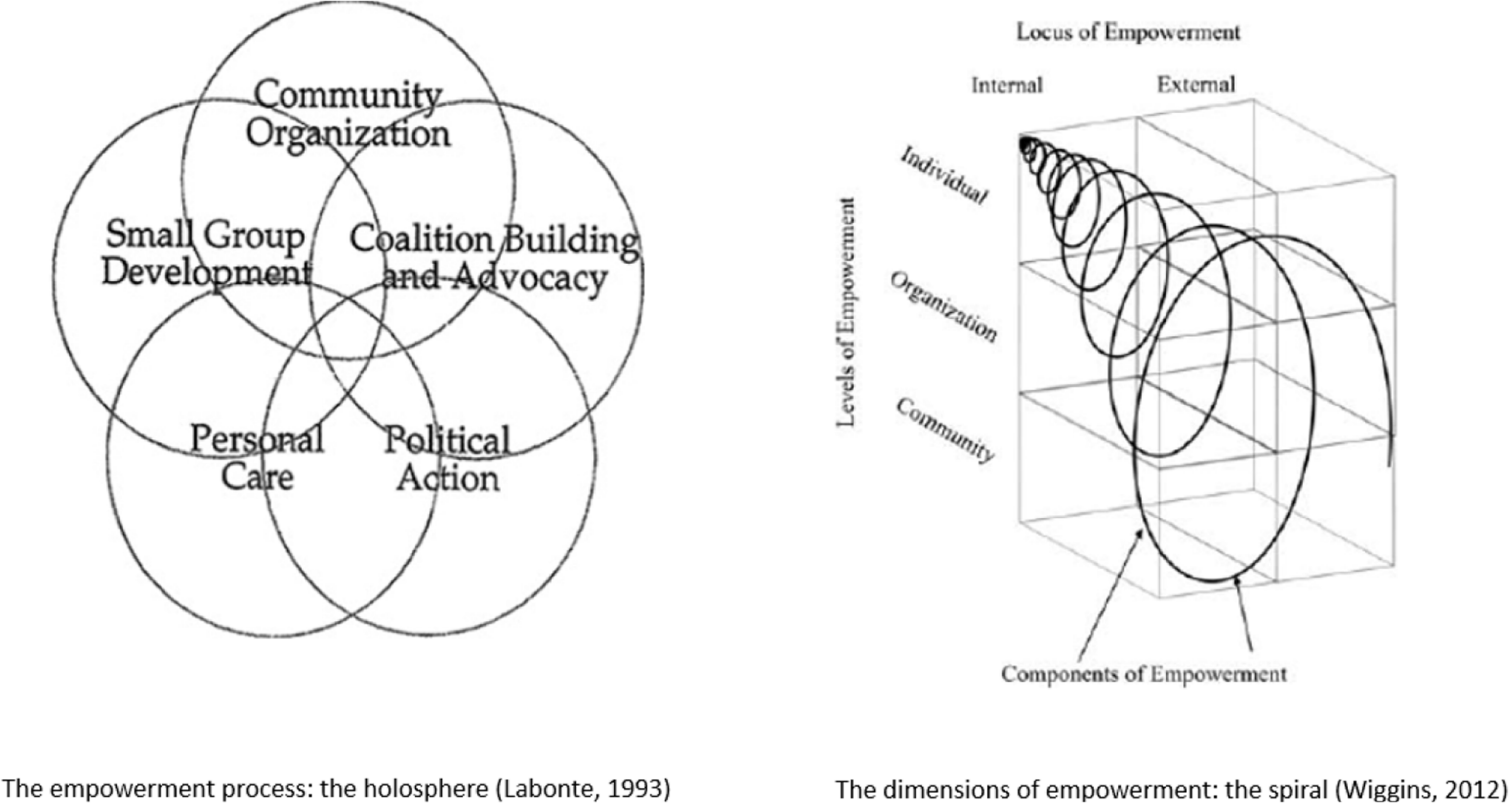
Complex representations of the dimensions of empowerment

These authors do not analyze collective empowerment as a phenomenon in itself but as a lever in health education programs [17, 18]. In contrast to Freirian principles [51], their approaches do not involve connecting communities with a critical analysis of cultures, economies, or power and belief systems that generate risky behavior or stigma in certain groups. They also overlook the collective ability to co-create knowledge as well as the power to act as a group to liberate themselves from sociopolitical constraints. In the end, these representations of collective empowerment remain complex (Fig. 1), and the distinctions between the organizational and community levels remain difficult to understand and measure [28, 52].

In addition to the inherent limits to the top-down approach, there are other considerations that justify a need for more studies from the community point of view. In the digital era, the proliferation of online patient groups makes it necessary to consider their empowerment a major social phenomenon that entails profound transformations in healthcare systems and society [53, 54]. Social media has a particular potential for collective empowerment due to its ease of access, horizontal structure, features, and real-time developments [55–58]. By enabling constructive interaction [45], social media helps shift the balance of power. For example, patients can demand alternative treatment options and even participate in their development. Despite the profound ways the internet has changed how people engage with their health, there are few studies of the empowerment of online patient communities.

#### A broader, bottom-up approach to the collective empowerment of online patient communities

Empowerment of online patient communities refers to both the individual empowerment of those who choose to join these organizations and the collective empowerment that characterizes the increased power, skills, knowledge, and influence of these communities. Their influence extends to how institutions operate as well as the political decisions that affect their members’ quality of life [59]*.* Up to now, very few studies have looked specifically at the collective empowerment of patients through social media (Table 2). Those who choose to join these communities are ordinary citizens facing a chronic illness, whether their own or that of a loved one. By joining a patient coalition, they feel they can better resolve their health problems and be in a better position to transform health and social institutions as a group [47].

Petric and Petrovčič [44] surveyed the members of about a hundred members of online forums, some of which were for patients. They identify the factors that enabled the combination of individual and collective empowerment: a sense of virtual community, engagement, and participation in the movement’s operations, actions, and projects. They highlight the importance of communication, defined as interactions between goal-driven engaged in discussions based on mutual understanding, sincerity, and cooperation. These factors improve not only the individuals’ social position but also help the group gain social power. Building on these reflections, Ammari and Schoenebeck [45] emphasize peer interactions within collective empowerment. They introduce the network empowerment model, which describes the transformation from a solitary search for information to a network process involving the formation of a group and the construction of collaborative sensemaking about illness. The authors highlight the role longstanding members play in creating community cohesion. Demjen [46] studies the content of online discussions and shows that humor is a vector of individual and collective empowerment. Self-deprecation can help lift taboos, create a feeling of belonging through certain code words, and bring the community together. To respond to the need for psychometric research, Atanasova and Petric [47] have built one of the first scales to evaluate collective empowerment of online patient communities, using two main dimensions and five complementary dimensions. The first dimension refers to drawing upon individual resources to collectively initiate change. The second involves coordinated collective actions that effect change. The five other dimensions are the sense of virtual community, engagement, intensity of engagement, civic participation, and offline emotional support. This research shows the budding social power within online patient communities and their impact on society.

The research summarized in Tables 1 and 2 approaches the collective empowerment of online patient communities from either a professional-centered approach focused on user relations [36–40] or a patient-centered perspective [44–47]. However, these studies do not take into account the creative and productive abilities of these communities, nor do they entertain the existence of priorities other than the goal of structural change.

The realm of healthcare has undergone profound changes that require new frameworks of analysis. First, patients have multiple identities. They are no longer simply recipients of care and treatments but also citizens, consumers, community members, co-creators of alternatives [60], and influential participants in the information society [61]. Moreover, patient collectives can design their own emancipation and overcome the lack of options or services by implementing their own services. This ability to obtain support and information, act, and cooperate has been augmented by the internet [62, 63].

To understand the growing power of these communities, this study looks at their organization as a self-contained system that is stable enough to continue even after some members leave [64]. The collective strength and intelligence of these groups exceed the sum of their members’ abilities [65, 66]. Until now, communities as collective subjects were rarely explored in the literature. The interest of this research thus lies in the analysis of collective empowerment from the point of view of patient communities and not as a result of interactions initiated by healthcare professionals or even the individual participation of community members.

## Methods and results

To gain a deeper understanding of the collective empowerment process of online patient communities, we carried out two complementary exploratory qualitative studies. The first, carried out with four experts, provides a general understanding of empowerment and allows us to model a process approach to be used as an analysis framework for the netnography that we carried out in the subsequent study of 21 forum participants.

### Qualitative study 1: preliminary study with experts

#### Methods

The first study is based on four semi-structured interviews with subject experts (a scientist and a journalists) and two knowledgeable laypeople (two patient organization managers) (Table 3).

**Table 3:** Sample of experts interviewed in study 1

Expert 1	Scientist, director of a laboratory recognized as a center of excellence
Expert 2	Doctor and science journalist in a major daily French newspaper
Expert 3	Patient representative holding a high-level position in a major patient organization (French Federation of Diabetics, AFD)
Expert 4	Patient representative holding a high-level position in a major patient organization (French Association against Myopathies - AFM – Téléthon)

Individual semi-structured interviews lasting 60–80 min were carried out from February to May 2015, using an interview guide including several themes (Additional file 1). Each expert provided a complementary vision of the issues involved and the means of empowering patients, thus ensuring a diversity in the points of view expressed.

The qualitative data analysis method is based on a manual thematic content analysis (intra- and inter-interview) [67] and derived from the Spiggle framework [68], which involves several distinct operations to organize data, extract meaning, and arrive at conclusions. Specifically, the first operation, categorization, consists of identifying a chunk or unit of data (theme) as belonging to, representing, or being an example of some more general phenomenon (process of classifying or labeling units of data). Categorization proceeded deductively (locating passages that represent a priori constructs, themes, or ideas based on the literature review – examples of deductive codes included “information need” and “peer interactions”) and inductively (identifying emergent categories from the data – examples of inductive codes included “community capital” and “co-development of innovative solutions”) [69]. In the second operation, called abstraction, previously identified groups are categorized into more general, conceptual classes. In our research, we distinguished four classes to comprehend the empowerment process (individual, identity, collaborative, and productive empowerment). The next steps that were followed (e.g., “comparison” and “dimensionalization”) made it possible to better understand the specific and distinctive nature of these different classes. At the end of the analysis process, the results thus contribute insights into the individual and collective needs and abilities of patients. They also provided a preliminary outline of the empowerment process.

#### Results

**Fig. 2:**
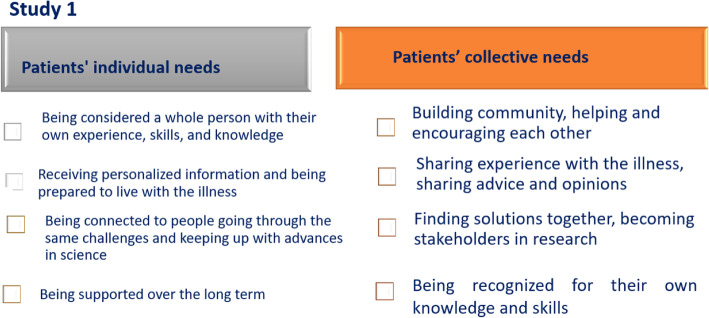
Patients’ individual and collective needs

The study carried out with these experts first allowed us to explore the diversity of patient needs (Fig. 2). Due to the chronic nature of their illness, patients undergo not only the impact of their treatment but also the constraints the illness imposes on their everyday lives and long-term plans. Their information needs are not limited to understanding the disease and its treatment; they need to understand the rules that will govern their new world. They have broad expectations, but the support provided is generally limited: “At the moment, we are much too centered on healthcare and not enough on health (expert 3).” In terms of the range of treatment options, it needs to be adjusted to the patient’s life plans: “They need a GPS…that can adapt the information to their situations…and to changes in their lives.” (expert 3). The chronically ill expect much from medical advances, but they learn that science, which is focused on highlighting innovation, tends to respond to other demands: “The goal isn’t to publish some good papers…but to find a medication that can cure people (expert 4).” The dissemination of scientific knowledge is hindered by its restriction to insider circles. Moreover, scientific discourse is being challenged by the public; health scandals have eroded trust in experts. Perhaps more surprising, the interviews pointed to the very widely expressed collective needs to build community, provide mutual assistance, and share experience with peers: “we see trust and empathy…these are discussions that are possible with people who have experienced misfortune. We understand each other.” (expert 3). Patients want to share opinions with each other, find solutions together, be stakeholders in the research and the evolution of practices, and be recognized for these contributions: “We are always there to poke them, push them, show them, ask them” (expert 4). By building a network based on the values of mutual aid and members’ resources (knowledge, skills, energy, time), enabling the development of innovative solutions, becoming stakeholders in research, and constantly prodding the experts, these communities assert themselves as the leading actors in collective empowerment.

**Fig. 3:**
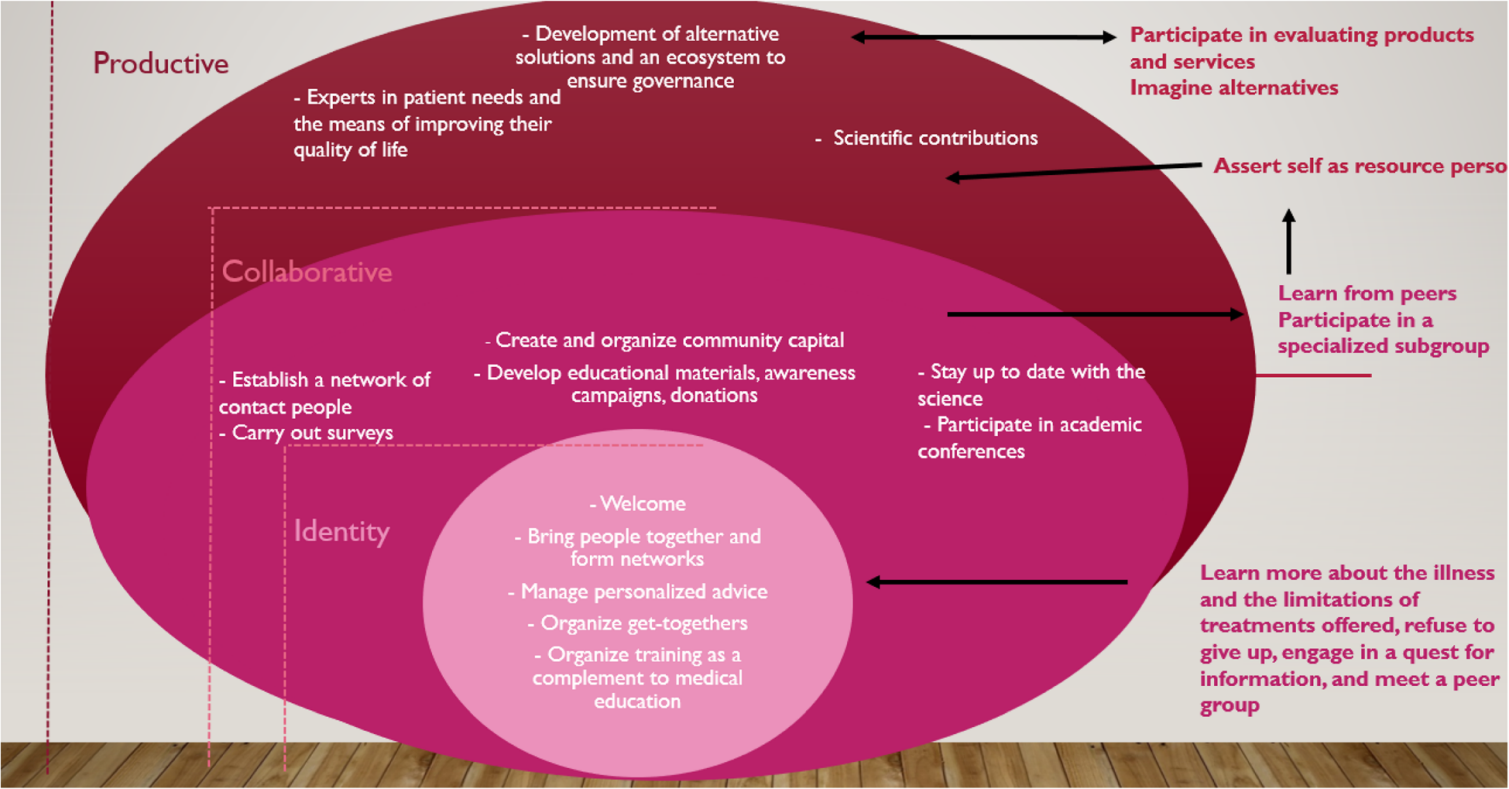
Individual and collective empowerment, a mutually beneficial process

Based on our interviews with these experts, we developed a preliminary process model of individual and collective empowerment (Fig. 3). Its development follows the path of chronically ill patients. These patients first express their troubles to their doctors and their loved ones. When they receive incomplete or inadequate responses, they feel frustrated. They refuse to give up but instead take charge of their destiny and begin a quest for solutions to the dysfunctions they are experiencing. This initiative marks their entrance into a process of empowerment that starts individually and becomes collective. These patients find peer communities on the web, ask them questions, and begin to see themselves as members of the group (identity empowerment). They discover the strength of this movement. Feeling understood and supported, they gain a sense of recognition and solidarity. At this point, these patients may decide to make a contribution to the collective project. Their individual or psychological empowerment [27] grows along with that of the group. The community grows along with its discussions. It creates its own resources (e.g., explanatory documents, social and legal aid) and thus builds community capital. This expansion marks the collaborative empowerment stage. Thanks to members’ reports on their experience, especially feedback on the quality of products and services, the group develops its expertise. As it hears the unsatisfied needs of patients and their loved ones, the network encourages its members and partners to co-develop alternate solutions, sometimes on a large scale, and develops ecosystems to maintain its governance; this is productive empowerment.

Thanks to their interactions with the group, new patients turn into connected contributors and enlightened actors. The community’s empowerment moves along through the broad categories of ability, identity, collaborative, and productive empowerment; because these levels are in fact phases, they can be transposed into a process. This preliminary representation of the collective empowerment process, stemming from four expert interviews, now needs to be strengthened, refined, and deepened through a second qualitative study.

### Qualitative study 2: Netnography of 21 forum members

#### Methods

By proposing an analysis framework making it possible to understand an online community’s empowerment through a three-dimensional process (identity, collaborative, and productive), the first qualitative study provided an especially useful basis for a second qualitative study carried out in the form of netnography. The primary objective of this second study is to verify the stages of empowerment and identify the individual determinants and organizational factors that enable the passage from one stage to another. The study was carried out in a French online patient forum called “Vivre sans thyroïde (Living without a thyroid)” (VST), one of the most documented and active patient groups in France in terms of messages exchanged. Dedicated to thyroid conditions, which affect one in 12 people,^2^ the forum distinguishes itself through its site architecture and educational features. The 485,000 messages on the site are categorized into 54,305 discussion threads organized into major topics that match the main thyroid conditions. The forum has 3800 visitors per day, mainly people having difficulty receiving diagnoses and/or treatment. The majority of these visitors only read the threads. The site statistics show that only 5–10% of users are registered and the remining 90–95% are not registered. The site is run by a volunteer team of moderators who provide guidance to posters, rank conversations, take down trolls, and delete disinformation.

The study took place in two stages: 8 months of immersion in the forum (May to December 2017) and 6 months specifically monitoring the progression of 21 participants (January to June 2018). Non-participant observation allowed us to become familiar with the modes of interaction among forum participants. This acculturation period was used to classify the types of discussion into the stages of empowerment identified in the first study and identify expressions and phrases related to each stage. This broad and intuitive exploration through quick reading involved thousands of participant discussions. The remarks and highlights were noted in a logbook. During the time of the study, the Levothyrox®^3^ crisis was occurring: a new formulation of a medication was brought on the market to substitute for an older treatment for thyroid patients. However, this medication led to a substantial number of negative side effects.^4^ The forum was overwhelmed with first uncertainty and then anger. We include this major event in this study.

After the immersion period, we continued the empirical phase by analyzing the messages of 21 actively engaged patients. To build this panel, we asked the forum’s president to designate about 30 of the most active participants in terms of recency, frequency, and quantity of messages sent, as in the customer segmentation method [70].^5^ We individually informed 30 participants about our research project and emailed them a request for permission to use their posts in our research. Twenty-one participants expressed their permission, thus satisfying the research ethics criteria defined by both Kozinets [32] and Convery and Cox [71]. These first email exchanges helped create an environment of trust between the researcher and the forum participants. We could thus precisely analyze their path within the forum, from their registration to their first message to their contributions as active members or moderators. These participants, who were recruited by the forum managers, act as teacher-facilitators to liberate and raise awareness, as described by Freire [51]. They support laypeople in a kind of digital apprenticeship and play a central role as facilitators, coaches, and specialists. Our study looks at key elements of their journey on the forum. They have participated on the forum an average of 8 years; the most veteran members began in 2004, while the latest arrivals came in 2017 (see Table 4). Our netnography provided elements to understand their engagement through an analysis of their personality traits. It also made it possible to link semantic markers to the evolution of the messages and each participant’s journey.

**Table 4:** Profile of the 21 forum participants studied

Individual	Gender	Sending date 1st message	Pathology	Number of messages	Authorization to analyze messages
**1**	M	25.3.2010	Hashimoto	5670	15 Jan. 2018
**2**	F	25.10.2011	Operation for benign nodules	2153	15 Jan.
**3**	F	3.3.2015	Laser nodule ablation	112	18 Jan.
**4**	F	9.8.2017	Hashimoto, cancer	990	15 Jan.
**5**	F	22.5.2008	Thyroid cancer	1010	17 Jan.
**6**	F	3.5.2004	Thyroidectomy	2993	27 Jan.
**7**	F	28.8.2012	Basedow’s disease	2208	15 Jan.
**8**	F	26.12.2008	Thyroid cancer	2351	29 Jan.
**9**	F	20.4.2007	Cancer	3203	15 Jan.
**10**	F	22.4.2015	Hashimoto	2279	16 Jan.
**11**	F	11.10.08	Cancer	5172	16 Jan.
**12**	F	16.4.2012	Thyroid cancer	1110	16 Jan.
**13**	F	5.10.2011	Hypothyroidism	810	15 Jan.
**14**	F	30.10.2009	Lobectomy	4773	15 Jan.
**15**	F	05.07.2004	Thyroidectomy, hypoparathyroidism	1558	27 Jan.
**16**	F	04.09.2017	Microcarcinoma	1369	18 Jan.
**17**	F	26.11.2007	Hypothyroidism	26,478	15 Jan.
**18**	F	22.3.2008	Thyroid cancer	2009	20 Jan.
**19**	F	10.9.2005	Thyroidectomy	1852	15 Jan.
**20**	F	5.12.2005	Hashimoto	1179	18 Jan.
**21**	F	26 .1.09	Thyroidectomy	620	19 Jan.

We carried out two complementary content analyses (thematic and lexical). The first was vertical, one forum participant at a time. After a scan of the 21 participants’ discussions, and based on the results of the first study (distinguishing individual, identity, collaborative, and productive empowerment), we divided the content of each participant’s messages into four categories. A chronological analysis showed similar progressions for all the participants studied. In the second step, a horizontal content analysis was carried out, grouping posts according to the stages of empowerment identified in the first study. To complement the manual thematic content analysis, lexical analysis software (Alceste) was used to identify the key words or textual units used that were representative for each category. These semantic markers revealed the participants’ communication intentions. The first category we identified involves the initial messages, in which the posters explain their reasons for joining the forum. The most frequent reasons were that they had just learned about their condition, they needed to have an operation, or their symptoms had gotten worse. The participants at this stage are very worried and tend to write long posts explaining their problems and emotions. The reference words were *I, nodule, problem, big, thyroid, norm, prescription, dose, symptom, you* (formal/plural). The second category contains messages written after the first post, when participants reformulate the individual responses they have received or clarify their questions so that the moderators have enough information to advise them. In these messages, they thank the other participants and the forum for their help. They appreciate being listened to, advised, and oriented. The reference words become *thank you, nodule, do, you* (informal). The third category includes the supportive messages the participants send to other forum members. These messages are posted when the new arrivals have been able to get past their own problems sufficiently to be able to take interest in the situations of other forum members who need support. At this point they become helpers more than askers, giving advice based on their experience. These posts also include the first messages in which the forum participants do not mention themselves at all but focus exclusively on supporting others. The semantic references become *you(informal), also, give, do, take, treatment, normal, hyper, courage, take care*. The fourth category includes messages the 21 forum members posted about the Levothyrox® crisis. These are messages warning about sudden declines in their health and their frustration that the authorities and the pharmaceutical company have been downplaying their problems. In these posts, members bring up the forum’s battles in the media and legal spheres. These messages bear critical analysis of the organization of treatment and the healthcare system. The reference words become *Levo, effect, formula, doctor, take, forum, change*.

By analyzing the messages of these forum members, it is possible to recognize the individual determinants that enable the passage from one empowerment stage to another, as well as the organizational factors underlying this evolution. As a result of this study, we establish a new model of collective empowerment that differs somewhat from the initial framework proposed in the first study. These results are presented in the following section.^6^

#### Results

##### Individual empowerment: the first step towards collective empowerment

Our results make it possible first of all to shed light on the motivations of patients who engage in the empowerment process. The first messages they send make their official entry into this process. By becoming forum members, the patients join a collective that has built a collaborative knowledge base [57] through thousands of conversations. In most cases, the new forum members summarize their situation and their treatment journey in their first post: “I have a cyst/nodule on the left lobe of my thyroid” (member 6), “…hyperplasia, adenomas, or nodules, what’s the difference?” (member 10), “I just got my results and I’m mystified” (member 13). These texts end with a crucial question about the members’ health. These requests for information are quickly answered, usually in less than 3 hours. The members are then initiated into their new occupation of patient. This apprenticeship begins with an educational dialogue with the moderator and then with other forum members. They take part in developing the skills and knowledge of the new arrivals, leading to psychological empowerment, which takes shape in a sense of autonomy, self-awareness, self-determination, and self-efficacy [27].

In the progression of the forum members we studied, we can identify a pivotal stage between individual and collective empowerment. This transition is rarely studied in the literature but is central to the community dynamic and essential for the renewal of the most active members. Unlike most users, who only post questions, active members stay on the site after finding their information. Why does their behavior different from those who register only to ask a question and then leave after expressing their thanks for the information received? After being supported upon their arrival, forum members who participate as volunteers over the long term feel responsible for paying it forward to new arrivals: “So I came looking for a little support and hope to be able to provide it to others once the situation has cleared up” (member 2). They are driven by a sense of gratitude, the feeling of never being able to pay back the support they have received. In the messages, “I” gradually becomes “we”: “We thyroid patients can go through a series of mental states that are difficult for us to manage…that’s why this forum is so valuable (member 11) They see their role as social inclusion agents [72], creating a feeling of belonging to a virtual society that welcomes, recognizes, empowers, and respects each member as a whole person: “being together and fighting together gives life meaning” (member 15). Now seasoned patients, these members become lead users who know how to solve problems empirically and develop alternative solutions [73, 74]. Their behavior is inspired by that of the forum’s founder, who personifies a charismatic leader [75], in her combined roles as guide, regulator, and mentor.

To approach the new arrivals, active forum members need to take a detached attitude to their own history. This mechanism evokes the notion of decentering [76]: forum members forget their own problems in order to focus on those of others and provide them guidance. They recognize the suffering of others as if it were their own and decide to provide as much support as they can: “I hope that when it’s my turn, the story of my adventures… can help boost the morale of others…and my own” (member 3). They are comfortable with their unique position: “We’re not here to judge others…just to make them think—give people the freedom to have discussions…That’s our responsibility as a forum!” (member 11). The choice to stay on the site is also motivated by a sense of reciprocity and altruism. These signs of consideration are expressed through empathetic attitudes and collaborative actions taken with no commercial or profit motivation [77, 78]*.* These feelings are closely linked to social skills, which involve the ability to socialize and have positive interactions with others like helping, sharing, cooperating, and comforting [79]. As they are able to contribute specific advice and search out information from their sources, the active forum members have special literacy abilities^7^ [81] and master new technologies: “As far as I know, the admission criteria for thermal ablation are…” (member 3), “I’d be surprised if you’re participating in the ESTIMABL protocol because you only take Cytomel after the operation” (member 8). This two-pronged knowledge, informational and technical, involves the ability to access medical information and understand, interpret, and use it to clear up health questions and then transform it into shared digital resources. These abilities can be seen in the composition of discussions, which involve an original mixture of solicitude, educational explanations, experience-based stories, and expressions of encouragement including emoticons. This mixture of empathy and knowledge transmitted in accessible language gives the forum its unique style.

By empowering others, these forum members are empowering themselves. This phase, which occurred with all of the forum members studied, takes place at different periods. The timeframe varies by personal characteristics (e.g., empathy or altruism) and also according to the seriousness of their condition, which allows them varying degrees of respite. For some, decentering is almost immediate: they may respond to another member’s question on the day they register. However, for others, this process may take several days or even more than a month; for one member, this took years (2 years 9 months). This forum would not exist without the central role of reciprocity [78] and altruism. On “Living without a thyroid”, these feelings combine with decentering and social skills and are enriched by literacy skills.

Now that we have identified the intermediate threshold that enables the progress from individual and collective empowerment as well as the psychological and cognitive features that determine the transformation from occasional poster to active forum member, we now continue by analyzing the different dimensions of collective empowerment.

##### The power of the collective

In the first qualitative study, we analyzed collective empowerment as a liberating process for vulnerable communities who organize to take charge of their destiny and assert themselves as a unified power confronting a frustrating system [3, 4]. We classified it into a three-dimensional process: identity, collaborative, and productive. This second, community-centered study refines this understanding and proposes a new three-phase sequence that is slightly different from the first model: 1) building a network around a shared identity (community empowerment); 2) building skills supported by actions (collaborative empowerment); 3) investing in social advocacy (societal empowerment). These steps mark the movement from an individual experience to the construction of a collective story, from searching for information to sharing knowledge, from the identification of a need to the co-development of innovative solutions, from individual frustration to political advocacy.

#### Community empowerment: building a network around a shared identity

The community empowerment stage involves the feeling of being “among ourselves” or “us together”. When it was created, VST presented itself as a forum offering information, help, and mutual assistance for thyroid patients. These foundations of identity and solidarity evoke what Wittgenstein [82] calls “family resemblance” based on “overlapping similarities of trust and mutual understanding.” The forum members share a collective identity in a convivial atmosphere. They call themselves “Levothyrians” or “butterflies” and speak of the “brotherhood of thyroid patients”, i.e., “Hello xxx and the butterfly crew” (member 21). In their dialogues, members spontaneously use familiar terms, usually as soon as the second message: “Hey there” or “take care”. The participants recognize the importance of this liberating space where they can give and receive support and critique the statements of the doctors and journalists who normally dominate the discourse on illness: “Who knows what I’m experiencing, my doctor or me?” (member 7) “Before I found this forum, I felt alone and down in the dumps” (member 13). To strengthen their bonds, forum members organize in-person get-togethers, “thyroid coffees”, or conferences on specific topics.

The forum is a place where patients can receive not only quick answers to urgent questions but also long-term emotional support throughout the course of their chronic conditions: “Everything I know about Hashimoto, I learned it on the forums…I’m going to print out the description on the site and bring it to my doc” (member 17) “Thanks so much to this awesome forum for all the super useful information on what was going on with me over the past few years” (member 4). Forum users make informed decisions based on the answers their peers provide. The thoughtful narratives and the diversity of comments enable awareness and perspective: “this is a question that I ask of myself and now I’m asking of you” (member 10).

The forum also plays the role of an outlet for expressing the social implications of the disease. Members come to “get it off their chest”: “It’s a disgrace not to let your patient know about negative side effects” (member 13), “…mistreated by doctors who have trouble telling the difference between this disease and nerve problems” (member 1). Feeling distressed and abandoned with their problems, the patients unite: “It warms my heart to read your message” (member 19), “being together and fighting together gives life meaning” (member 15).

On VST, almost all the posts lead to interactions: individual questions, announcements about healthcare reforms, or sharing of media coverage focused on thyroid patents. The topics are commented on in recurring communication loops. These conversations create a sensemaking system that helps participants reach a shared and intersubjective understanding of their situation [57]. The forum’s internal organization into thematic sections helps bring members with similar concerns together. This closeness gives rise to initiatives that generate pragmatic solutions, fertile ground for collaborative empowerment.

#### Collaborative empowerment: skill building supported by actions

Collaborative empowerment refers to the ability to self-organize in order to compensate for the dysfunctions of treatment regimens by taking advantage of the many contributions made by forum members. When offered services that do not meet their expectations, these patients are in a better place to propose corrective measures or devise more appropriate solutions. By bringing together initiatives based on member contributions and partnerships developed with exterior actors, certain pages of the forum appear to have become a living lab. The forum has produced many achievements, such as an informal directory of recommended doctors, symptom measurement scales co-developed by the patients, a chart with dosage equivalents for a medication, clear explanations of the benefits and disadvantages of a new treatment from a patient’s point of view, a list of questions to ask the surgeon, and even the best directions to a ward in a pavilion-style hospital. This “community capital” is similar to collaborative consumption, defined by sharing practices based on non-ownership of goods and services and engagement in shared activities [83].

Other initiatives are carried out in collaboration with companies or outside experts. The co-development process at work here evokes a concept derived from service-dominant logic [84] in which consumers design part of the product but do not necessarily maintain control of it. In this way, forum members collaborate with various stakeholders: a laboratory, researchers, independent public entities, and pharmacists in bordering countries. Among the achievements we identified are presentations at scientific conferences and the creation of an app based on a monitoring chart the patients developed.

Some suggestions from forum members have not yet been developed but could someday become promising initiatives, such as “launching a clinical study of whether dosages should be modified in the winter/summer” (member 15) or the suggestion to “make thyroid conditions a medical speciality in itself, given the substantial number of patients affected” (member 2).

Certain features of the forum help make knowledge more accessible: a search engine and an FAQ. Others contribute to the creation of knowledge (e.g., the ability to take a poll or add to the glossary) or help create relationships (e.g., member directories or a schedule of people having operations).

In parallel with the participative production of resources, the forum has also positioned itself as a social advocacy force, especially during the Levothyrox crisis. This scandal made very clear the sharp imbalance of power between the patients, the pharmaceutical lab, and the healthcare establishment.

#### Societal empowerment: transforming the world

Societal empowerment is characterized by a constructed, structured, and massive resistance [85] and a political positioning of demands. During the Levothyrox® crisis, the forum became the nerve center of the patients’ combat. To mobilize its members and build a case based on participants’ testimony, it capitalized on its experience in community and collaborative empowerment.

Community empowerment can be seen in the sympathetic response to the distress of the patients in over 1000 discussion threads: “I’ve been taking the new Levo for 4 months now…I’m getting worse and worse; no more energy” (member 15) “I’m disgusted…it took me a year to find my thyroid setpoint—one year of trial and error and suffering, and now here we go, back to square one!” (member 16). Their messages express their frustration with the lack of attention paid to their concerns: “These people own the rights to our life and death—it’s as cynical as that!” (member 4) “If they hadn’t brushed off our objections and left patients suffering helplessly, we wouldn’t be here now” (member 7). The rumblings from the web [32, 86] and the responses of the authorities, perceived as inadequate, exacerbated the tensions.

At the peak of the crisis, posts denounced the lobbies and failures at the highest level and were sometimes peppered with expressions of revolt against “Big Pharma” and the “big switch forced on patients” (member 9). The patients felt that no matter their state of health, they were forced to accept what was given them because one pharmaceutical company had the monopoly on this medication in the French market. The impossibility of a boycott strengthened the feeling “of being trapped by a manipulative company interested only in profit” (member 13) “I’m suffering because of a mistake I didn’t make; no-one is assuming any responsibility, much less apologizing!” (member 16) “We should be able to choose our medications according to the company’s ethics. If you think about it…they’d be nothing without us” (member 20).

Collective empowerment led to concerted actions like internal polls; an emergency collaboration with European pharmacies to arrange a supply of Euthyrox®, which had become very difficult to find in France; and the crowdfunding of a lawsuit. The forum got public opinion involved and dominated the media. It launched petitions, organized local and national actions, and activated international networks to compare the French situation to that of other countries. It was able, among many other things, to write its media messages within just a few hours and alert both the health authorities and the press.

The empowerment became societal when the feelings of injustice, resentment, and failure of democratic principles were shared by a large number of people, creating a wave of social exasperation: “It’s not enough to just sit in your room and complain; you have to get yourself seen and recognized” (member 10). Their rightful anger [87] was channeled into a media and legal battle against the establishment and the pharmaceutical company. Established as an advocacy force, VST took the matter to court. The forum thus made good on its mission to “produce discernment” and its right to know the truth, in this case the reasons why the formula was changed. It advocated that scientific pronouncements should no longer be the sole source of legitimacy. It demanded that patients be allowed to participate in all the studies of the Medicines Agency (ANSM), including its research work. And it denounced the dominant position one pharmaceutical company was allowed to have.

The forum’s civic action [88] is testimony to the strong impact of a united and responsive community determined to make changes in institutions and the market.

**Fig. 4:**
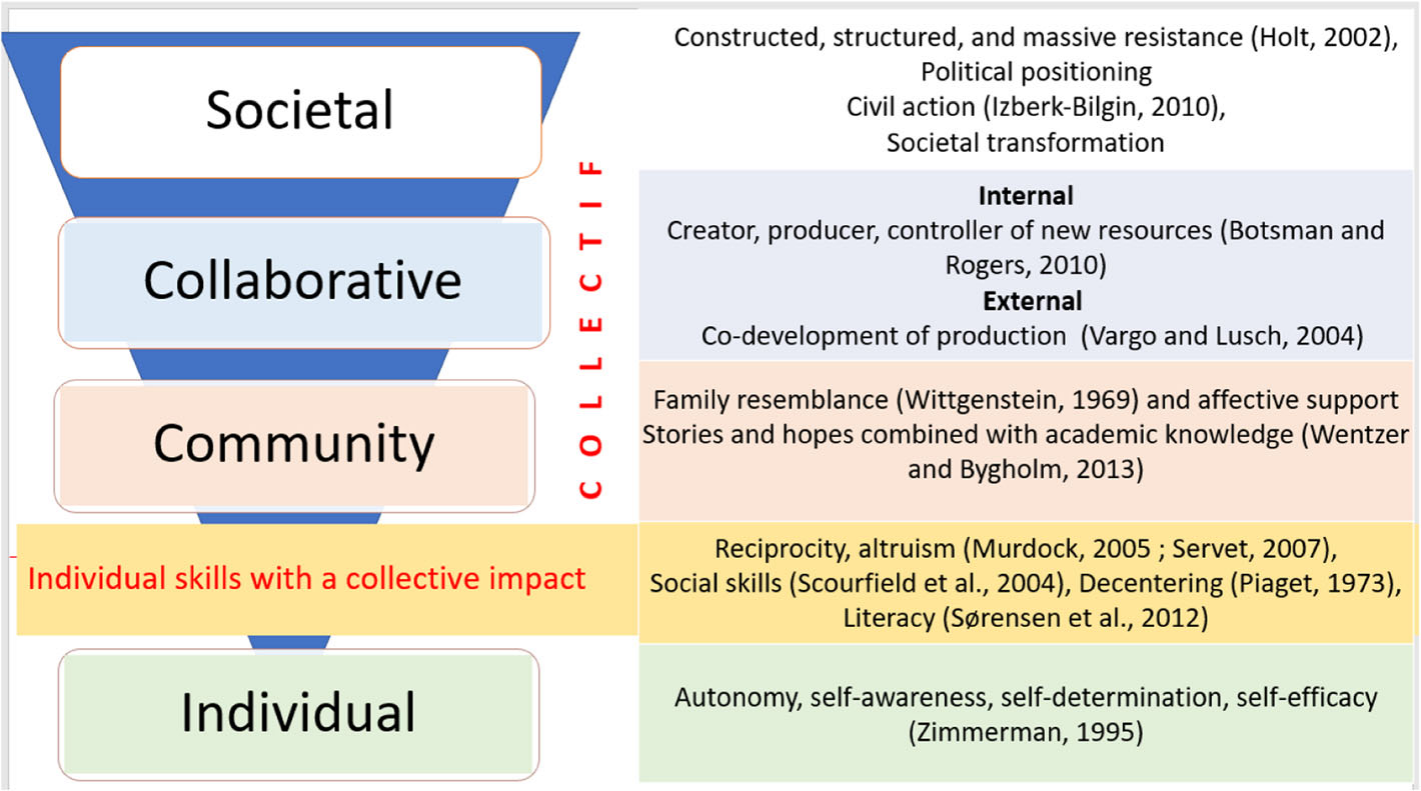
The empowerment of chronic patients, a 4-step process

Based on the results of this second study, we can propose a new empowerment process model (Fig. 4).

**Fig. 5:**
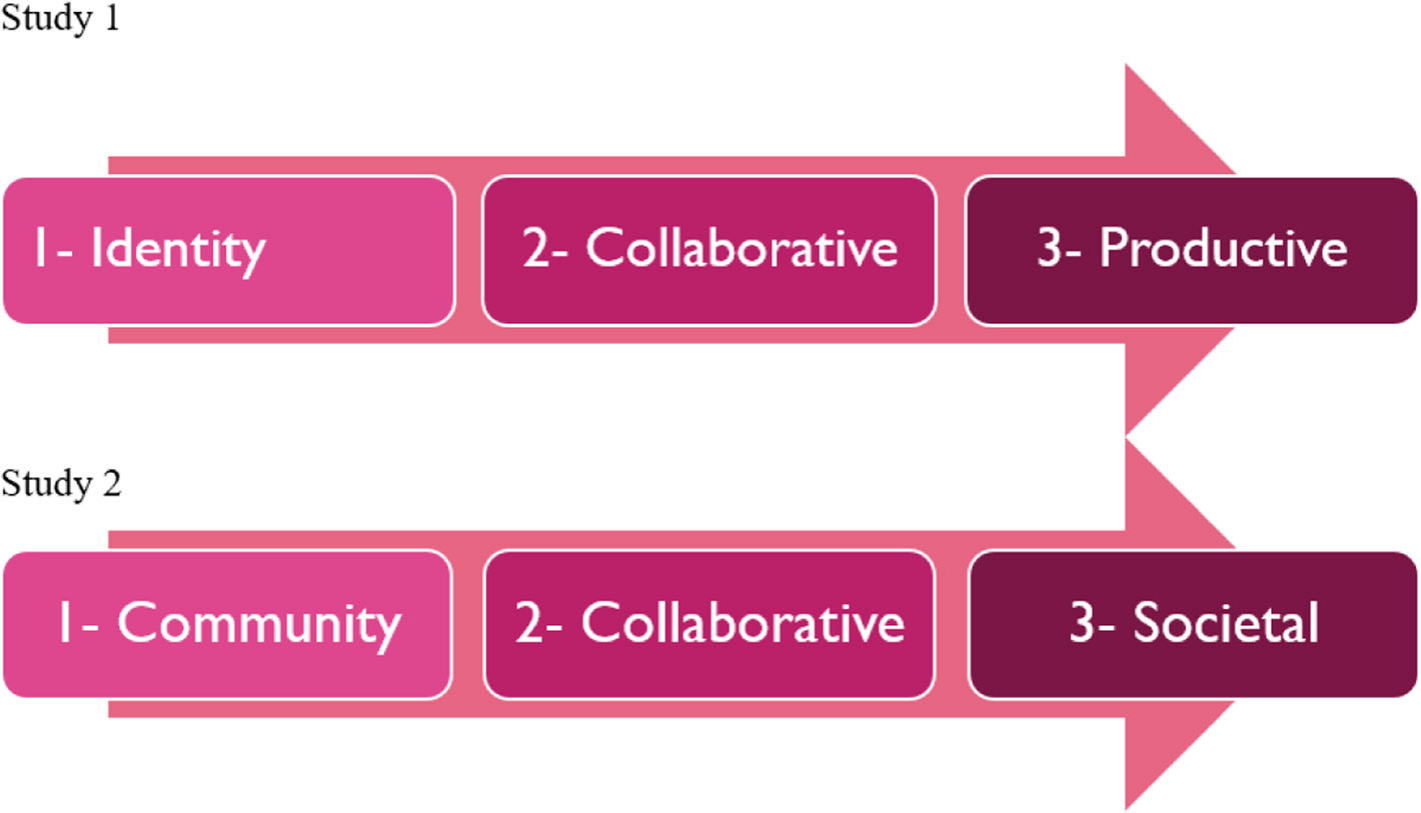
Comparison of the three stages of collective empowerment identified in studies 1 and 2

When the results of study 1 with experts are compared to study 2 with patients (Fig. 5), we see that the first two stages of the collective empowerment process are quite similar except for one name: the “identity” level is now the “community” level to emphasize the strong and structured interactions. On the other hand, the third stage is different: in the first case it is called “productive” because it relates to finding treatment solutions or adequate long-term support; in the second case it is “societal” due to the sociopolitical nature of patient demands and the civic struggle brought to court. It thus appears that the final stage differs due to the priorities and major causes identified by the actors involved (scientists, patient organization leaders, patient community members).

## Discussion

### Key takeaways

This healthcare management study examines the collective empowerment of patients from a process and community-centered perspective. It is thus meant to build on previous research that has mostly looked at empowerment as an outcome (psychological empowerment) [26, 28] and taken a top-down [1] and individual-centered approach to patients (29–31). More specifically, our study takes into account four new phenomena that influence patients’ relationship with the healthcare system: 1) the change of status from recipient of healthcare to citizen/consumer/influencer, 2) the growth of the social web, 3) peer apprenticeship in the occupation of chronic patient, and 4) the transformative power of these movements. This study integrates these changes to make a significant contribution to the understanding of the collective empowerment of patients online. As such, it complements previous professional-centered [36–40] and patient-centered approaches [44–47]. More specifically, both empirical studies propose a new reading of this multi-level construct, first by considering individual empowerment to be the first step to collective empowerment. We then identify the levels of collective empowerment: identity/community, collaborative, and productive/societal. Finally, we show that these dimensions adapt to the priorities of the movement. This study also provides information about the individual determinants that enable the transformation of a novice to a resource person who is both empowering and empowered.

### Theoretical implications

Since we found a lack of literature in health services management regarding these online patient communities and their collective power, and because of their growing role in healthcare system performance [5–7], this research offers a better understanding of the complex nature of patients’ collective empowerment and the process underlying it from the point of view of patient communities [33, 34, 43]. Specifically, through a conceptualization of the process dynamics of collective empowerment, it offers first an in-depth understanding of this construct’s components by highlighting the importance of the creative and productive abilities of these communities (productive empowerment), and the collective oppositional force they generate (societal empowerment). Second, our research reveals both the stability of the sequence of stages in the empowerment process (individual empowerment ➔ identity / community empowerment ➔ collaborative empowerment) and the adaptability of the final stage (productive empowerment / societal empowerment). Thirdly, by analyzing the criteria for progression through the empowerment process, we shed light on the unmet needs and motivations that drive chronically ill patients to engage in an individual and then collective empowerment process through social media.

### Practical implications

In the operational sphere, this study advocates for an increased recognition of the role of online communities and forums in the lives of patients. They have filled in not only the missing link between general practitioners and specialists but also the services lacking before and after care, all while participating in the dissemination and personalization of medical knowledge. These platforms put active listening and educational discussions at the heart of their actions. They also serve as an alert system in the event of a healthcare crisis. The most active contributors keep track of news and innovations, which generate many comments and even critical analyses and civic debate. An official certification process for forums that are rigorously and ethically moderated could thus lead to increased recognition of their contributions within the healthcare system. The empowerment process we have shown here also calls for a more dynamic reconsideration of the patient’s role. By considering the criteria and semantic markers we have identified, it is possible to observe the journeys of novice patients who gradually become engaged volunteers and then lead users equipped with knowledge, skills, and a vast network. As stakeholders with a unique experience and a wide audience, these experienced patients can be called upon by healthcare actors to co-develop service offerings and organizations, healthcare sectors and establishments, innovative research programs, or health policy. More broadly, it seems essential for healthcare professionals to keep up with this kind of social media so that they can tap into the patients’ collective intelligence and alternative visions and better understand patient concerns before these escalate into anger. Finally, this research highlights the central role of the most active forum members in the management of online patient communities. The appeal, energy, and influence of these platforms seem to depend strongly on these central members’ contributions and activities. Thus, by providing reference elements to help identify these members (in terms of both individual attributes and types of post), this study can facilitate the support of these patients in the development of their collective empowerment and the establishment of a relational approach aimed at building loyalty among these active contributors.

### Limitations and future directions

Despite its contributions, this research has some limitations that could become avenues of future research. First, this netnography only covered one forum and analyzed the activity of only 21 active patients. The robustness of the collective empowerment process model could be increased through new qualitative research carried out among a larger number of patients with different conditions and on different social media platforms like Facebook, Instagram, or Twitter. Second, a measuring instrument could be developed using a quantitative approach in order to evaluate the various gradual facets of collective empowerment. Moreover, this study focused on the point of view of the patient community. It thus raises the question of empowering practices in medical teams. In a relational framework of patient communities and healthcare professionals, it seems useful to take a dyadic approach to explore how healthcare personnel working in a frequently tense social context view the emergence of this more independent group that disrupts their habits and hierarchies. In order for patients’ individual and collective empowerment not to be experienced as an additional constraint but rather an opportunity to rethink collaborative treatment strategies and new organizational models, it is necessary to create the conditions for a similar empowerment process for healthcare teams [89].

Finally, a study could be carried out on the impact of patients’ societal empowerment. This could evaluate the extent to which the institutions and private companies implicated in patient protests make changes to their organization and regulatory frameworks. A study like this could provide information about their resilience strategies and ability to reinvent themselves.

## Conclusions

The main contribution of this study is its new model of collective patient empowerment as a dynamic process centered around various types of gradual phases. The identity/community phase (level 1) indicates a period when a shared identity is defined among peer patients through a collective narrative. The collaborative dimension (level 2) is defined by selfless participation in a pragmatic co-development of assets and resources, including emotional or informational support and innovations that help improve patients’ daily lives. The final phase can be productive or societal (level 3). The productive form of collective empowerment involves a search for solutions requiring significant investments and logistics. Empowerment on a societal scale is reached when individual frustrations aggregate into social indignation and citizen demands encroach into the sociopolitical sphere. By showing the components of a liberating movement that transforms individual fragility into collective strength, this research sheds new light on communities’ contribution to patient empowerment and the healthcare system.

## Supplementary Information

**Additional file 1.** Interview Guide.

## Data Availability

The datasets used during the current study are available from the corresponding author upon reasonable request.

## Footnotes

1 Patient question on the *Vivre sans thyroïde* (Living without a thyroid) forum: https://m.forum-thyroide.net/phpBB/ftopic36356-15.html, accessed 09/12/2020.

2 Source: American Thyroid Association - https://www.thyroid.org/media-main/press-room/#:~:text=Prevalence%20and%20Impact%20of%20Thyroid,are%20unaware%20of%20their%20condition. Accessed 3 oct. 2020.

3 Levothyrox® is the new formulation of a synthetic thyroid hormone prescribed as a treatment to replace natural thyroxine when the thyroid can no longer produce sufficient quantities.

4 Over 17,000 reports in 8 months (April–November 2017), according to the pharmacovigilance report 2 published on 1/30/2018: https://www.forum-thyroide.net/pdf/Levothyrox_Position_VST_CS_No_3_20180206.pdf. Accessed 22 December 2020.

5 *Recency*: date of last purchase. When we transpose this criterion into the patient community, it becomes the last login date. *Frequency*: The number of consecutive purchases within a given period (week, month, year). In the case of the forum, this meant looking at the number of consecutive logins since the person registered. *Purchase amount*: The cumulative amount of the purchases made by the customer. For the forum, this meant looking at the quantity of text produced.

6 To strengthen the reliability of our results, we submitted the first version of our analysis of the results to participants and the President of the “Vivre sans thyroïde” forum. We gave them a month to provide their feedback. Five participants (out of a total of 21) and the President shared their comments with us. We took their remarks into account and integrated them all into the final document.

7 “The degree to which individuals have the capacity to obtain, process, and understand basic health information and services needed to make appropriate health decisions” (Ratzan and Parker, 2000, cited in Kindig et al. [80]).
