# Supplementary material for: Collective empowerment of an online patient community: conceptualizing process dynamics using a multi-method qualitative approach
Source: BMC Health Serv Res. 2021 Sep 13;21:958. doi: 10.1186/s12913-021-06988-y (PMC8436557; doi:10.1186/s12913-021-06988-y)
Supplement: Supplementary file 1 — Additional file 1. Interview Guide. [file 12913_2021_6988_MOESM1_ESM.docx]

Appendix A

**Interview Guide**

In the introduction, the aim of the interview  (to better understand the individual and collective empowerment of chronic patients) is presented.

Then, each of the following themes is discussed:

**Theme 1**: Specific features of the healthcare relationship for the chronically ill

**Theme 2**: Specific needs of chronic patients, especially in regard to health information

**Theme 3**: Relationships between the scientific knowledge of healthcare teams and the experiential knowledge/expertise of patients

**Theme 4**: Different forms of patients’ individual and collective involvement/commitment

**Theme 5**: Patients communities’ impact on the health system and research
